# How Does Feeding Development and Progression onto Solid Foods in PKU Compare with Non-PKU Children During Weaning?

**DOI:** 10.3390/nu11030529

**Published:** 2019-02-28

**Authors:** Sharon Evans, Anne Daly, Jo Wildgoose, Barbara Cochrane, Satnam Chahal, Catherine Ashmore, Nik Loveridge, Anita MacDonald

**Affiliations:** 1 Birmingham Women’s and Children’s Hospital NHS Foundation Trust, Birmingham B4 6NH, UK; a.daly3@nhs.net (A.D.); satnam.chahal@nhs.net (S.C.); catherine.ashmore@nhs.net (C.A.); anita.macdonald@nhs.net (A.M.); 2 Bradford Teaching Hospitals NHS Trust, Bradford BD9 6RJ, UK; jo.wildgoose@bthft.nhs.uk; 3 Royal Hospital for Children Glasgow G51 4TF, UK; Barbara.cochrane@ggc.scot.nhs.uk; 4 Danone Early Life Nutrition, Macquarie Park, New South Wales 2113, Australia; nik.loveridge@danone.com

**Keywords:** Phenylketonuria, protein substitute, weaning, feeding development

## Abstract

Weaning is complex for children with phenylketonuria (PKU). Breastmilk/infant formula and phenylalanine (Phe)-free infant protein-substitute (PS) are gradually replaced with equivalent amounts of Phe-containing food, a semi-solid/spoonable weaning PS and special low-protein foods. In PKU, feeding patterns/practices during weaning in PKU have not been formally evaluated. In this longitudinal, prospective, case-control study (*n* = 20) infants with PKU transitioning to a second-stage PS, were recruited at weaning (4–6 months) for a comparison of feeding practices and development with non-PKU infants. Subjects were monitored monthly to 12 months and at age 15 months, 18 months and 24 months for: feeding progression; food textures; motor skill development and self-feeding; feeding environment; gastrointestinal symptoms; and negative feeding behaviours. Children with PKU had comparable weaning progression to non-PKU infants including texture acceptance, infant formula volume and self-feeding skills. However, children with PKU had more prolonged Phe-free infant formula bottle-feeding and parental spoon feeding than controls; fewer meals/snacks per day; and experienced more flatulence (*p* = 0.0005), burping (*p* = 0.001), retching (*p* = 0.03); and less regurgitation (*p* = 0.003). Negative behaviours associated with PS at age 10–18 months, coincided with the age of teething. Use of semi-solid PS in PKU supports normal weaning development/progression but parents require support to manage the complexity of feeding and to normalise the social inclusivity of their child’s family food environment. Further study regarding parental anxiety associated with mealtimes is required.

## 1. Introduction

The dietary management of the inherited disorder of protein metabolism, phenylketonuria (PKU), particularly in infancy and childhood, is a critical component of care, and treatment is recommended to commence prior to 10 days of age [1]. The feeding of young children with PKU is complex, with most children requiring a severe to moderate natural protein restriction (commonly < 10 g/day), in order to control blood phenylalanine (Phe) levels within target ranges and prevent severe intellectual impairment [2]. In infancy, the diet is supplemented with a Phe-free protein substitute formula to meet energy and non-Phe protein requirements. Maintaining adequate intakes of energy, total protein equivalent, and natural protein to individual tolerance in PKU is important for the promotion of normal growth and development and maintaining acceptable Phe control. 

Weaning defined as the introduction of solid or more textured foods into an infant’s liquid-based diet, is a crucial time as it has an impact on future food preferences and longer-term health status. Feeding patterns and practices during weaning in PKU have never been formally evaluated and compared with the normal population. Ideally weaning in PKU should reflect the weaning process of healthy non-PKU infants [3], aiming to transition from an exclusively liquid diet, to a mixed diet encompassing a wide variety of foods, tastes and textures; and encouraging development of independent self-feeding. 

There is anecdotal information about how best weaning should be approached in this population: for example, administering a protein substitute in the form of a paste or spoonable protein substitute to young children appears to be well accepted, with fewer associated feeding problems [4], but no prospective, controlled study of the benefits of such products has been carried out. In the UK, it has become practice to introduce a Phe-free weaning protein substitute that is administered as a low volume semi-solid texture, given by spoon and suitable for use from weaning age. The amount of weaning protein substitute is gradually increased, and the volume of Phe-free infant formula reduced during the weaning period. Retrospective data suggests that most refusal of weaning protein substitute is generally associated with normal weaning behaviour, commonly occurring, alongside food refusal, during teething and illness [5]. Nevertheless, the timing of introduction of a second stage protein substitute is vital, with early (<17 weeks of age) or late (>26 weeks of age) weaning and delayed introduction of more textured foods increasing the likelihood of refusal of both weaning protein substitute and food in general [5].

Compared with the general population, there are additional issues and anxieties for parents/carers of children with PKU, particularly with respect to the acceptance and tolerance of Phe-free protein substitutes and special low protein foods. It is possible that earlier weaning (from 17 weeks) may reduce food neophobia and improve acceptance particularly of low protein foods but this has not been proven in PKU. 

These disorder-specific challenges are an adjunct to the ‘general’ tasks of weaning infants such as: ensuring the safe and timely introduction of solid foods around key developmental milestones [6], supporting age appropriate growth rates [7], the support of oral motor and speech development and normal feeding and swallowing [8], maintaining nutritional adequacy [9], and supporting social and cultural competence and enjoyment of eating.

This multi-centre longitudinal, prospective case-control study aims to evaluate the weaning development of infants with PKU transitioning to a semi-solid Phe-free protein substitute and to compare the feeding patterns, practices and difficulties with those of non-PKU infants during weaning.

## 2. Materials and Methods

### 2.1. Subjects

In this study, all infants meeting the inclusion criteria (i.e., diagnosed with PKU via newborn screening with no illness or co-morbidities who were commencing weaning and required a second stage Phe-free protein substitute during the 2-year study period) were recruited from 3 UK inherited metabolic disease centres: Birmingham Children’s Hospital, Bradford Teaching Hospitals and the Royal Hospital for Children, Glasgow. Subjects were recruited by the one dietitian in each centre. 

Non-PKU control children who were commencing weaning, were matched with the PKU group for mother’s educational level and birth order; they were recruited by one of two dietitians. Control children included infants from a local Birmingham community centre and siblings of children with inherited metabolic disorders. Control subjects proceeded with weaning according to their own experience, or advice from health visitors/health professionals as required. This was independent of the study procedures.

### 2.2. Study Design

Subjects were recruited when solid foods were introduced (age determined by parental discretion, usually between 4–6 months) and monitored monthly to 1 year of age and then at 15, 18 and 24 months of age (total of 10–12 visits).

### 2.3. Feeding Development Diary

Prior to each monitoring visit, all parents/carers completed a non-validated qualitative 3-day diary recording: stage of feeding progression with respect to food textures (thin puree, thick puree, weaning foods, or normal foods); motor skill development (ability to use utensils and drink from a cup/straw) and self-feeding; the feeding environment including where infants were fed (e.g., highchair, lap, table); and any gastrointestinal symptoms (mouth ulcers, constipation, diarrhoea, colic, retching, vomiting, flatulence, burping, abdominal pain/distention). For the PKU group, the frequency of taking protein substitute and the incidence of 10 different behaviours associated with administering protein substitute (spitting it out, closing mouth, crying at start, crying at end, turning head away, pushing spoon away, deliberately spilling, refusing more than they take or refusing as much as they take) was also recorded over 3 days at each monitoring visit. These records were cross checked with parents/carers by the dietetic researcher at each visit.

### 2.4. Weaning Protein Substitute and Weaning Diet

At each monitoring visit, daily intake of weaning protein substitute, infant protein substitute and natural protein (breastmilk, standard infant formula or solid food equivalent) were recorded (PKU group). The weaning protein substitute (Anamix First Spoon, Nutricia Ltd, Trowbridge, UK) was a powdered Phe-free supplement containing essential and non-essential amino acids, carbohydrate, fat, vitamins, minerals, trace elements and long chain polyunsaturated fatty acids. When mixed with water it produced a low volume, semi-solid spoonable paste. Dose and increments of weaning protein substitute, infant protein substitute and natural protein intake are described in another publication [10]. 

After the infants with PKU had been established on low protein weaning foods (such as puree fruit or vegetables), weaning protein substitute was introduced. Usually 5–10 g of weaning protein substitute powder (providing 2–4 g protein equivalent) was introduced once daily, and gradually built up to 3 doses per day. This coincided with a stepwise protein equivalent reduction in infant protein substitute as well as gradual introduction of Phe-containing weaning foods to replace the Phe equivalent from breast milk or standard infant formula. Although any change/decrease in breastmilk or standard infant formula was at the request of parents/carers, the nutritional aim was to provide a combined total of 3 g/kg/day of protein [2] from protein substitute (both infant and weaning) and natural protein exchanges (from food, breastmilk or standard infant formula).

### 2.5. Ethical Approval

This study was conducted according to the guidelines laid down in the Declaration of Helsinki and favourable opinion was given by the local research ethics committee (West Midlands–South Birmingham). Written informed consent was obtained from parents/carers for all children. 

### 2.6. Data Analysis

Subject numbers were based on the number of new PKU diagnoses meeting the study criteria across 3 centres during the 2 years study period. Descriptive statistics and quantitative outcome measures were summarised. Non-parametric tests (Wilcoxon signed rank and Mann Whitney tests) were used to compare PKU and control groups for differences in quantitative measures using GraphPad Prism version 6.01 for Windows, GraphPad Software, La Jolla California USA. 

## 3. Results

### 3.1. Subjects

Twenty infants (diagnosed with PKU on newborn screening from Birmingham Children’s Hospital (*n* = 17), Bradford Teaching Hospitals (*n* = 2) and the Royal Hospital for Children, Glasgow (*n* = 1) who were commencing weaning and required a second stage Phe-free protein substitute during the study period were recruited (Table 1).

Twenty control children commencing weaning and matched with the PKU group for mother’s educational level (*n* = 18) and birth order (*n* = 18), were also recruited. Two control children were of Caucasian/Afro Caribbean origin, all other subjects were white Caucasian. 

### 3.2. First Weaning Foods and Textures

Parents/carers in both PKU and control groups decided the age to commence weaning and this differed between groups (median PKU: 4.1 m (range: 2.8–5.0); control 5.0 m (range: 3.7–6.0); *p* = 0.001 (Mann Whitney)). Children with PKU commenced weaning on a narrow range of low protein foods –mostly puree fruit, vegetables or low protein rusks; whilst control children had a larger variety of first weaning foods with most consuming baby rice/porridge (Table 2). The texture of first weaning foods was similar between groups. 

There was no significant difference between the groups in terms of age of progressing onto different textured foods other than the PKU group starting thin fluids at a younger age, but this is associated with the younger age of weaning in this group (Table 3).

### 3.3. Fluid Intake

Total fluid intake from breastmilk and formula (combined protein substitute and standard infant formula for PKU) was similar in both groups at all ages (Table 4). Breastmilk intake was estimated based on recommended quantities of infant formula for that age. Children with PKU consumed less fluid each day (mean difference 82 mL/day; range 13–184 mL) than did control children up to 12 months of age, thereafter, control children consumed less (mean difference 72 mL/day; range 35–113), but this was not statistically significant (Mann-Whitney). Fewer children with PKU were breastfed (*n* = 3) compared with control children (*n* = 9) during the study period. Children with PKU also stopped infant formula (Phe-free) on average later than control children. By 12 months of age all children with PKU had fully transitioned to eating solid foods as the source of their phenylalanine/protein exchanges in place of breastmilk or standard infant formula, with most (*n* = 18) completing this transition by 8 months of age. However, protein substitute formula continued to provide a significant portion of energy, protein and nutrient intake during the gradual transition onto the weaning protein substitute. During this transition period, low protein milk replacements were introduced to meet energy requirements. In comparison, nearly half (45%; *n* = 9) of the control children had transitioned from breastmilk or standard infant formula to cow’s milk by 12 months of age, and all but one (95%; *n* = 19) by 24 months of age.

### 3.4. Independent Self-Feeding Skills

Children with PKU were more likely to be spoon fed by the parent and drink from a bottle compared with control children and this was still apparent at 2 years of age. They were also slower to start drinking from a cup or straw than control children (Table 5). In terms of age of developing specific self-feeding skills, there was no significant difference between the groups other than for self-finger feeding which commenced at a younger age in control children (Table 6).

### 3.5. Feeding Environment

At 4 months of age, both groups were more likely to feed children in a bouncer followed by a highchair or their parent’s lap (Table 7). Feeding in a bouncer was more common in the control group from 4–7 months than in the PKU group. From 6 months of age, a highchair was the most common place to feed for both groups until 24 months when the control group were more likely to be fed at the family table. 

### 3.6. Frequency of Meals and Snacks

Children with PKU generally had one less meal or snack of solid food per day compared to control children and sometimes an additional meal of infant (Phe-free) formula; although by 2 years of age this difference was not evident (Table 8). 

### 3.7. Negative Behaviour with Protein Substitute (PKU Group)

**Figure 1:**
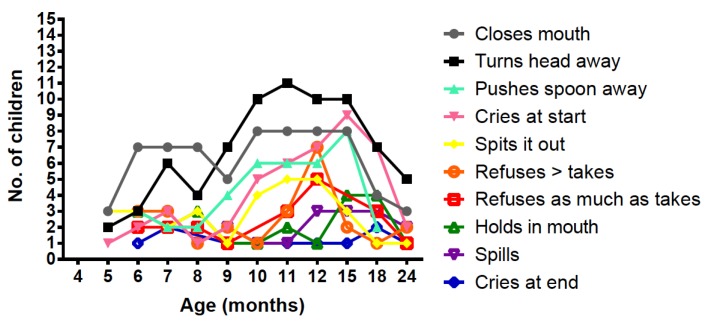
Number of children displaying different negative behaviours associated with protein substitute administration at each age.

The most frequent negative behaviours associated with taking weaning protein substitute were closing the mouth or turning the head away when offered the protein substitute; occurring in at least 5 children at each monitoring visit. These negative behaviours were most common at the age of 10–18 months coinciding also with the age for teething (Figure 1).

### 3.8. Gastrointestinal Symptoms

**Figure 2:**
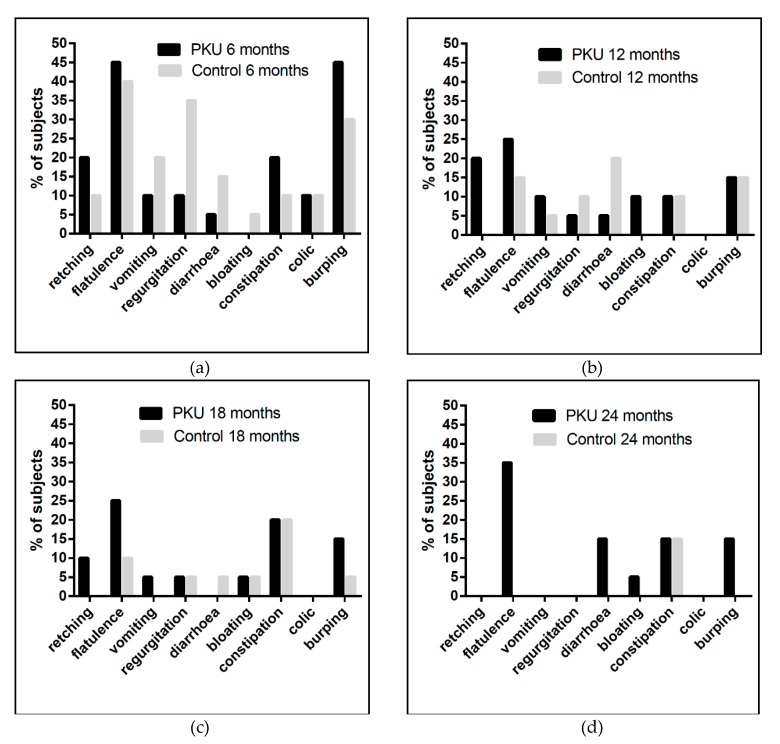
Percentage of subjects reporting gastrointestinal symptoms at (**a**) 6 months, (**b**) 12 months, (**c**) 18 month and (**d**) 24 months of age.

There was no significant difference between the two groups for total number of incidences or number of subjects experiencing vomiting, diarrhoea, bloating, constipation or colic at any age. However, more children with PKU reported problems with flatulence (0.0005), burping (*p* = 0.001) and retching (*p* = 0.03) at all ages; and more controls reported regurgitation (*p* = 0.003) (Wilcoxon signed rank test). Symptoms generally declined with increasing age although vomiting and diarrhoea were variable, most likely associated with illness (Figure 2).

## 4. Discussion

Children with PKU in this cohort generally experienced very similar weaning experiences to their non-PKU peers. Progression onto more textured foods, reduction in fluid (breastmilk/infant formula) intake, self-feeding skills, the feeding environment and gastrointestinal symptoms such as diarrhoea, vomiting, constipation and colic were similar across the two groups and where differences did occur, most had resolved by 2 years of age.

There may be a critical period between 6–7 months when infants are developmentally ready for the introduction of more textured foods, and delaying this beyond 10 months of age may increase the risk of food refusal, fussy eating, delayed self-feeding, poor routines, unhealthy eating, and limited variety in the diet [6,11]. This is particularly important in PKU where the diet is already quite limited and feeding problems are known to be more common [12]. The stepwise introduction of a semi-solid Phe-free protein substitute and the stepwise decrease in liquid protein substitute during weaning enabled age-appropriate progression with respect to food textures, in line with the general aims of weaning. This permitted children to become accustomed to solid foods with less dependence on high volumes of infant protein substitute; and it appeared to maintain the appetite for solid foods by reducing energy intake from protein substitutes. 

Typically, during weaning, non-PKU children gradually increase solid food intake and concomitantly reduce breastmilk/formula intake, generally replacing it with cow’s milk at 12 months of age. For children with PKU the process is more complex; reducing infant protein substitute formula results in a reduction in energy intake that is not compensated for by the lower energy weaning protein substitute. This energy deficit is made up by the introduction of a low protein milk replacement and low protein special foods, and these are generally introduced before 12 months of age. 

Total volume of fluid intake was similar in both groups up to 11 months of age (except at 4 months when subject numbers were much smaller as some had not commenced solids). By 12 months, half the control children had fully transitioned to cow’s milk, but it was another 6 months before half the PKU group had fully transitioned to weaning protein substitute and low protein milk replacement. Children with PKU relied on their infant protein substitute and subsequently continued to drink from a bottle for longer in order to meet energy requirements. However, by 2 years of age most children in both groups had stopped both Phe-free infant formula and breastmilk/standard infant formula. Fewer infants with PKU were breastfeeding (in combination with infant formula (Phe-free) than controls at weaning commencement, but it has been established in other studies that the duration of breastfeeding is short in PKU, and this is an area that warrants further research to examine any barriers [13]. Breastfeeding can successfully be incorporated into the PKU diet, but a normal intake of breastmilk would exceed the Phe requirements of infants, and thereby lead to poor blood Phe control. Therefore the volume is limited, carefully controlled, and given in combination with a Phe-free infant formula. During weaning, any breastmilk is gradually replaced with the equivalent quantity of Phe/protein from a food source. Although the timing of this process is led by parents, the gradual introduction of protein containing foods is important to help the feeding development of a child particularly with respect to adjusting to new tastes and textures. This process requires close supervision by the metabolic dietitian. 

Children with PKU were given first non-milk foods 1 month earlier than their non-PKU peers and the range of first weaning foods was unsurprisingly less varied than in control children, due to the protein content of some of the more common first weaning foods, such as baby rice, other cereals and mashed potato. However, the age for moving onto more textured foods and developing self-feeding skills was similar. Children with PKU were more likely to be spoon fed by a parent for longer than controls although this may reflect the need to give the weaning protein substitute and to ensure the full amount of prescribed natural protein, given as measured Phe exchanges, are consumed. 

Self-finger feeding occurred at a later age in children with PKU possibly associated with longer spoon feeding by parents, but they also generally consume one less meal or snack per day than controls. However, children with PKU were less likely to be offered snacks between meals in case this decreased appetite for their Phe-free weaning protein substitute. 

Control children were more likely to be fed at the family table from 15 months of age compared with children with PKU. This is consistent with previous reports that suggest children with PKU are more likely to be fed separately from the rest of the family [14]. Parents of children with PKU may feel the need to be more in control of their child’s mealtime due to fears of poor metabolic control; so, eating at a separate time to the family and spoon-feeding may help parents to concentrate and focus more on their child’s eating. In addition, there may be an element of guilt surrounding the different foods offered to their child with PKU compared with the rest of the family, and this requires further study.

Gastrointestinal symptoms were different in both groups with flatulence, retching and burping more common in the PKU group and regurgitation in controls. Parents perceived flatulence as being directly associated with weaning protein substitute and this may be associated with its low volume and high osmolality. Negative behaviours associated with weaning protein substitute also occurred during food consumption around the age of 10-18 months and coinciding with teething. The problems observed were comparable to a previous retrospective report [5]. 

## 5. Conclusions

Weaning development and progression in children with PKU is comparable to that of control children, despite the additional changes required in terms of introducing protein exchange foods, and weaning protein substitute. Where differences occur, they are likely to be associated with additional parental anxiety, as food intake needs to be controlled and closely monitored. This may have led to extended spoon-feeding, earlier cessation of breastfeeding, prolonged bottle feeding with Phe-free infant protein substitute and separate mealtimes to the rest of the family; which may in turn delay independent self-feeding. Parents require support to manage the complexity of feeding times, and to normalise the social inclusivity of their child’s food environment. Further study about parental anxiety associated with mealtimes is required. 

## Figures and Tables

**Table 1:** Subject demographics.

	PKU					Control				
Subject No.	Sex	Age at Weaning (months)	PKU Siblings	Mother’s marital Status	Age Stopped Breast Feeding (mths) *	Sex	Age at Weaning (months)	PKU Siblings	Mother’s marital Status	Age Stopped Breast Feeding (mths) *
1	M	4.1		Married		M	4.6	3	Married	
2	M	5.0		Married		F	5.0		Married	10
3	F	3.3		Single		M	5.5		Married	9
4	M	3.4	1	Married		F	4.4		Married	7
5	F	5.0		Partner		M	5.7		Married	8
6	F	3.7		Partner		M	5.8		Married	
7	M	3.9		Married		M	6.0		Single	
8	F	5.0		Married		M	3.8		Partner	
9	F	4.5		Single		M	5.7		Partner	
10	M	2.8		Single		F	3.7		Single	
11	M	3.7		Married	5	F	4.5		Single	
12	F	3.7		Married		M	4.0		Partner	12
13	M	3.3		Married		F	5.0		Partner	
14	M	4.7		Married	8	F	4.5		Partner	18
15	M	4.4	2	Married		F	5.0		Married	
16	M	4.4	2	Married		M	4.5		Married	
17	M	4.5		Married		M	5.0		Partner	
18	M	5.0		Partner		M	4.4		Married	7
19	M	4.0		Married		F	5.0		Married	10
20	M	4.0		Married	8	M	5.0		Married	18

* age stopped breast feeding for those still breast feeding at point of weaning (3–6 months).

**Table 2:** First weaning foods and textures.

	PKU %	Control %
**1st Weaning Foods**		
Baby rice/porridge	0	75
Puree fruit	85	55
Puree vegetables	65	30
Rusks- regular	0	25
Low protein rusks	45	0
Mashed potato	5	20
Stage 1 baby food jar	0	20
**Weaning Textures**		
Thin puree	70	70
Thick puree	60	45
Mashed	15	25
Finger food	5	15

**Table 3:** Average age for progression onto different textured foods.

	Mean Age in Months (Range)		
	PKU	Control	*p* value *
Thin puree	4.5 (3–6)	5.0 (3–8)	0.03
Thick puree	5.8 (3–7)	5.4 (3–8)	0.29
Weaning foods	5.2 (4–8)	5.3 (4–7)	0.82
Normal family foods	7.6 (5–11)	7.1 (4–10)	0.46

* Mann-Whitney.

**Table 4:** Total fluid intake (mL) per day; PKU vs. control.

	Mean Volume ml (Range)						
Age (m)	PKU Phe-Free Infant Formula	*n*	PKU Standard Infant Formula	*n*	PKU Total Volume	Control Total Volume	*n*
**4**	493 (160–870)	11 *	304 (170–540)	11 *	796 (400–1170)	981 (750–1260)	9 *
**5**	510 (190–900)	20	260 (90–480)	20	744 (435–1200)	776 (550–1110)	18 *
**6**	509 (210–720)	20	181 (60–340)	20	623 (450–750)	663 (450–840)	20
**7**	567 (300–840)	19	126 (600–380)	11	641 (360–930)	653 (240–1000)	20
**8**	511 (200–810)	19	90 (90–90)	2	521 (200–810)	614 (270–900)	20
**9**	498 (250–840)	19	90	1	503 (250–930)	559 (285–1010)	20
**10**	397 (240–600)	19	90	1	403 (240–600)	485 (120–890)	20
**11**	387 (110–640)	18	90	1	393 (110–640)	501 (180–900)	17
**12**	347 (80–840)	18	0	0	347 (80–840)	502 (240–1160)	11
**15**	448 (210–840)	13	0	0	448 (210–840)	488 (270–600)	6
**18**	311 (120–840)	10	0	0	311 (120–840)	383 (300–450)	4
**24**	420 (240–750)	4	0	0	420 (240–750)	540	1

* not all subjects had commenced weaning at this age; m: months.

**Table 5:** Age of subjects for attaining feeding skills (%) (*n* = 20 PKU; *n* = 20 Control).

	PKU Control	4 m *n* = 13 *n* = 11	5 m *n* = 19 *n* = 20	6 m *n* = 20 *n* = 20	7 m *n* = 20 *n* = 20	8 m *n* = 20 *n* = 20	9 m *n* = 20 *n* = 20	10 m *n* = 20 *n* = 20	11 m *n* = 20 *n* = 20	12 m *n* = 20 *n* = 20	15 m *n* = 20 *n* = 20	18 m *n* = 20 *n* = 20	24 m *n* = 20 *n* = 20	*p* value ^#^
Spoon fed by parent	PKU Control	100 89	100 100	100 100	100 100	100 100	100 100	100 95	100 95	100 85	95 80	90 55	85 25	0.008
Spoon feeds self	PKU Control	8 0	11 6	10 5	15 15	5 10	20 5	10 30	30 25	50 40	65 55	80 85	100 90	0.29
Fork feeds self	PKU Control	0 0	0 0	0 0	0 0	0 0	0 5	0 5	0 5	15 5	10 40	40 60	80 85	0.25
Finger fed by parent	PKU Control	0 44	21 39	40 50	65 40	55 50	60 70	70 75	70 85	65 70	50 65	60 25	35 5	0.78
Finger feeds self	PKU Control	5 15	15 40	45 80	90 85	100 90	100 95	100 95	95 100	100 100	100 100	100 100	100 90	0.76
Drinks from bottle	PKU Control	100 67 *	95 61	100 75	95 70	100 100	100 100	100 85	90 90	85 95	90 80	100 65	85 30	0.006
Drinks from cup	PKU Control	8 0	16 17	30 35	40 55	45 70	55 75	60 85	65 85	65 90	80 95	85 95	85 90	0.004
Drinks from straw	PKU Control	0 11	0 6	0 5	0 5	0 10	10 20	10 25	15 25	30 30	30 40	45 50	70 70	0.002

* *n* = 3 breastfed only (no bottles); ^#^ Wilcoxon signed rank test; m: months.

**Table 6:** Mean age (months) of feeding skills development.

	Mean Age in Months (Range)		
	PKU	Control	*p* value *
Self finger-feeding	6.5 (4–8)	5.7 (4–9)	0.01
Self spoon-feeding	12.8 (5-24)	12.6 (5–18) 1 still not	0.99
Self fork-feeding	19.2 (12–24) 3 still not	18.1 (12–24) 2 still not	0.41
Drinking from a cup	8.8 (4–15) 2 still not	7.8 (5–15)	0.43
Drinking from a straw	14.7 (9–24) 6 still not	14.9 (4–24) 4 still not	0.94

* Mann-Whitney.

**Table 7:** Percentage of children fed in different environments at different ages.

		4 m	5 m	6 m	7 m	8 m	9 m	10 m	11 m	12 m	15 m	18 m	24 m
Highchair	PKU Control	38 11	53 33	70 60	80 90	85 85	85 90	80 95	90 100	100 100	80 80	70 75	50 45
Lap	PKU Control	31 56	26 28	30 25	15 20	10 10	10 20	15 20	15 20	5 0	10 5	15 0	5 0
Bouncer *	PKU Control	38 67	26 67	15 40	0 20	5 5	5 5	5 5	0 5	5 0	5 0	5 0	0 0
Table/Booster **	PKU Control	0 0	0 0	0 0	0 0	5 10	0 5	0 0	0 0	0 0	5 25	15 40	30 85
Other (sofa, cot, pushchair, floor)	PKU Control	8 11	21 6	0 10	10 15	10 5	15 10	15 10	5 0	5 0	5 10	10 10	15 5

* bouncer = a low-lying chair that can be used to lightly bounce babies, it is designed to fully support a baby in a semi reclined position; ** booster = a seat that can be placed on an existing seat to ‘boost’ the height of the child at a normal table; m: months.

**Table 8:** Median number of meals or snacks per day.

**Total Number of Meals/Snacks**												
	**4 m**	**5 m**	**6 m**	**7 m**	**8 m**	**9 m**	**10 m**	**11 m**	**12 m**	**15 m**	**18 m**	**24 m**
PKU	6	6.5	6	6	6	6	6	6	5	6	5	6
Control	9	8	7	8	7.5	7	7	7	7	7	6	6
*p* value *	0.01	0.07	0.37	0.01	0.02	0.07	0.02	0.06	0.04	0.03	0.12	0.80
**Number of Solid Meals/Snacks**												
PKU	2	3	3	3.5	3	3.5	4	4	4	4	4	4
Control	1	2	3	4	5	5	5	5	5	5	5	5
*p* value *	0.05	0.27	0.57	0.38	0.30	0.01	0.02	0.04	0.09	0.02	0.03	0.16
**Number Fluid Breastmilk/Formula Feeds**												
PKU	6	6	5	5	4	4	3	3	2	2	1	0
Control	7.5	5	4	4	4	3	2.5	3	2	0	0	0
*p* value *	0.005	0.22	0.04	0.33	0.80	0.12	0.05	0.76	0.12	0.06	0.04	0.25

* Mann-Whitney; m: months.

## References

[1] Van Spronsen F.J., Van Wegberg A.M., Ahring K., Bélanger-Quintana A., Blau N., Bosch A.M., Burlina A., Campistol J., Feillet F., Giżewska M. (2017). Key European guidelines for the diagnosis and management of patients with phenylketonuria. Lancet.

[2] Smith I., Cockburn F., Barwell B., Brenton D., Chapple J., Clark B., Curzon G., Davidson D., Heeley A., Laing S. (1993). Recommendations on the dietary management of phenylketonuria. Report of Medical Research Council Working Party on Phenylketonuria. Arch. Dis. Child..

[3] British Nutrition Founation Introducing Solid Foods to Your Baby. https://www.nutrition.org.uk/healthyliving/nutrition4baby/complementaryfeeding.html.

[4] Macdonald A., Daly A., Davies P., Asplin D., Hall S.K., Rylance G., Chakrapani A. (2004). Protein substitutes for PKU: What’s new?. J. Inherit. Metab. Dis..

[5] Evans S., Daly A., Macdonald J., Pinto A., Macdonald A. (2017). Fifteen years of using a second stage protein substitute for weaning in phenylketonuria: A retrospective study. J. Hum. Nutr. Diet..

[6] Agostoni C., Decsi T., Fewtrell M., Goulet O., Kolaček S., Koletzko B., Michaelsen K.F., Moreno L., Puntis J., Rigo J. (2008). Complementary Feeding: A Commentary by the ESPGHAN Committee on Nutrition. J. Pediatr. Gastroenterol. Nutr..

[7] Vail B., Prentice P., Dunger D.B., Hughes I.A., Acerini C.L., Ong K.K. (2015). Age at Weaning and Infant Growth: Primary Analysis and Systematic Review. J. Pediatr..

[8] Stevenson R.D., Allaire J.H. (1991). The Development of Normal Feeding and Swallowing. Pediatr. Clin. N. Am..

[9] Alles M.S., Eussen S.R., Van Der Beek E.M. (2014). Nutritional Challenges and Opportunities during the Weaning Period and in Young Childhood. Ann. Nutr. Metab..

[10] Evans S., Daly A., Wildgoose J., Cochrane B., Chahal S., Ashmore C., Loveridge N., MacDonald A. (2019). Growth, protein, and energy intake in children with PKU taking a weaning protein substitute in the first 2 years of life: A case-control study. Nutrients.

[11] Northstone K., Nethersole F., Emmett P. (2001). The effect of age of introduction to lumpy solids on foods eaten and reported feeding difficulties at 6 and 15 months. J. Hum. Nutr. Diet..

[12] Macdonald A., Harris G., Rylance G., Asplin D., Booth I.W. (1997). Abnormal feeding behaviours in phenylketonuria. J. Hum. Nutr. Diet..

[13] Pinto A., Adams S., Ahring K., Allen H., Almeida M., Garcia-Arenas D., Arslan N., Assoun M., Altınok Y.A., Barrio-Carreras D. (2018). Early feeding practices in infants with phenylketonuria across Europe. Mol. Genet. Metab. Rep..

[14] Macdonald A., Rylance G., Asplin D., Hall K., Harris G., Booth I. (1994). Feeding problems in young PKU children. Acta. Paediatr..

